# A combined CaMKII inhibition and mineralocorticoid receptor antagonism via eplerenone inhibits functional deterioration in chronic pressure overloaded mice

**DOI:** 10.1111/jcmm.15355

**Published:** 2020-06-23

**Authors:** Helen E. Driessen, Magda S. Fontes, Leonie van Stuijvenberg, Maike A. Brans, Marie‐Jose Goumans, Marc A. Vos, Toon A. van Veen

**Affiliations:** ^1^ Division of Heart & Lungs Department of Medical Physiology University Medical Centre Utrecht Utrecht The Netherlands; ^2^ Department of Cell and Chemical Biology LUMC Leiden The Netherlands

**Keywords:** CaMKII, contractile performance, echocardiography, heart failure, mineralocorticoid receptor antagonism, patchy fibrosis, strain analysis

## Abstract

In the diseased and remodelled heart, increased activity and expression of Ca^2+/^calmodulin‐dependent protein kinase II (CaMKII), an excess of fibrosis, and a decreased electrical coupling and cellular excitability leads to disturbed calcium homeostasis and tissue integrity. This subsequently leads to increased arrhythmia vulnerability and contractile dysfunction. Here, we investigated the combination of CaMKII inhibition (using genetically modified mice expressing the autocamtide‐3‐related‐peptide (AC3I)) together with eplerenone treatment (AC3I‐Epler) to prevent electrophysiological remodelling, fibrosis and subsequent functional deterioration in a mouse model of chronic pressure overload. We compared AC3I‐Epler mice with mice only subjected to mineralocorticoid receptor (MR) antagonism (WT‐Epler) and mice with only CaMKII inhibition (AC3I‐No). Our data show that a combined CaMKII inhibition together with MR antagonism mitigates contractile deterioration as was manifested by a preservation of ejection fraction, fractional shortening, global longitudinal strain, peak strain and contractile synchronicity. Furthermore, patchy fibrosis formation was reduced, potentially via inhibition of pro‐fibrotic TGF‐β/SMAD3 signalling, which related to a better global contractile performance and a slightly depressed incidence of arrhythmias. Furthermore, the level of patchy fibrosis appeared significantly correlated to eplerenone dose. The addition of eplerenone to CaMKII inhibition potentiates the effects of CaMKII inhibition on pro‐fibrotic pathways. As a result of the applied strategy, limiting patchy fibrosis adheres to a higher synchronicity of contraction and an overall better contractile performance which fits with a tempered arrhythmogenesis.

## 1 INTRODUCTION

During cardiovascular pathology, molecular changes can gradually affect cardiac function, eventually leading to heart failure. For the heart to function properly, it needs sufficient electromechanical coupling between cardiomyocytes via gap junctions (in the ventricles mainly connexin 43, Cx43), substantial excitability of cardiomyocytes through the sodium channel (Nav1.5) and appropriate architectural stability provided by extracellular collagen type I and III. In the diseased and remodelled heart, the activity and expression of Ca^2+^calmodulin‐dependent protein kinase II (CaMKII) is elevated. This, together with an excess of fibrosis, and a decreased electrical coupling and cellular excitability, leads to reduced cardiac function via disturbed calcium homeostasis and architectural tissue integrity.

CaMKII is activated by binding of Ca^2+^/calmodulin (CaM) to the regulatory domain leading to a conformational change exposing the catalytic domain of CaMKII. During episodes of a sustained high cytosolic Ca^2+^ concentration, CaMKII can become constitutively active via autophosphorylation.^1^ Its targets involve various ion channels, with emphasis on calcium handling proteins within the cardiomyocytes. For example, phosphorylation of the L‐type calcium channel leads to slower inactivation, phosphorylation of phospholamban leads to dissociation from SERCA2a thereby increasing Ca^2+^ re‐uptake in the sarcoplasmic reticulum, while phosphorylation of the ryanodine receptor leads to more systolic Ca^2+^ release and diastolic Ca^2+^ leak.^2^ Although CaMKII initially initiates beneficial effects, sustained activity of CaMKII eventually triggers pro‐arrhythmic electrical remodelling and leads to transcription of pro‐hypertrophic genes inducing structural remodelling eventually leading to heart failure (HF). Therefore, CaMKII is considered as an interesting drug target in HF therapy.^3^, ^4^

Approaches with acute CaMKII inhibition appeared anti‐arrhythmic in numerous animal studies. Indirect inhibition of CaMKII with W7 suppressed almost all torsade de pointes (TdP) in chronic AV block dogs (CAVB).^5^ W7 also prevented methoxamine‐induced TdPs in rabbits, improved conduction velocity (CV) and reduced arrhythmogenicity in isolated perfused rabbit hearts.^6^, ^7^, ^8^ Furthermore, in various rat and mouse studies the anti‐arrhythmic potential of acute CaMKII inhibition was also observed (reviewed in Ref. 9). With regard to contractile performance, acute CaMKII inhibition in mice subjected to 15 days of transverse aortic constriction (TAC) showed significant restoration of function.^10^ This was also found in mice which underwent TAC for 3 weeks and received pharmacological inhibition of CaMKII during the last week.^10^ Furthermore, genetic deletion of CaMKII δ showed beneficial effects on function and structural remodelling after six weeks of pressure overload.^11^, ^12^ However, another study showed that chronic CaMKII inhibition preserved conduction characteristics and Cx43 expression, but lacked anti‐arrhythmicity and did not preserve function in mice that were subjected to long‐term TAC (namely 16 weeks). This was most likely the result of persistent fibrosis formation eventually disrupting the architectural integrity of the myocardium during this extended period of TAC.^7^ Chronic CaMKII inhibition in the latter study was achieved using transgenic mice possessing cardiac‐specific expression of autocamtide‐3‐related‐peptide (AC3I, AC3I‐mice) which inhibits a conserved region of the CaMKII regulatory domain.^13^ These studies suggest that CaMKII inhibition at least party is effective in restoring normal conduction parameters but not completely reverts adverse cardiac remodelling in the long term and ultimately does not prevent heart failure.

A previously conducted study in our laboratory, interestingly, showed that fibrosis, a major player in adverse cardiac remodelling, could be depressed in physiologically aged mice by antagonism of the renin‐angiotensin‐aldosterone‐system. Long‐term administration of losartan (angiotensin‐receptor blocker) and eplerenone (aldosterone‐receptor blocker), showed equal effectiveness.^14^ Aldosterone binds to its cytosolic mineralocorticoid receptor (MR) which is known to have pro‐fibrotic downstream targets (reviewed in Ref. 15). The MR can translocate to the nucleus and facilitates transcription of transforming growth factor beta 1 (TGF‐β1), fibronectin and collagens.^16^ Additionally, MR signalling can induce cell (trans)differentiation and proliferation of (myo)fibroblasts.^17^ Therefore, MR antagonism via eplerenone, a second‐generation selective MR antagonist, could potentially prevent fibrosis formation during pathological cardiac remodelling.

Here, we investigated the combination of CaMKII inhibition together with eplerenone treatment as strategy to prevent cardiac remodelling, and subsequent functional deterioration leading to heart failure in an AC3I‐mouse model of long‐term chronic pressure overload.

## 2 MATERIALS AND METHODS

### 2.1 Animals

Mice with cardiac‐specific expression of AC3I were kindly provided by Dr Anderson, The University of Iowa, USA. AC3I mice were bred in a C57BL/6 background. To investigate the additional effect of eplerenone on top of chronic CaMKII inhibition, 14 AC3I mice received eplerenone treatment (200 mg/kg/d) for a period of 11 weeks starting seven days after TAC (group name AC3I‐Epler). These mice were compared to 16 AC3I mice not receiving eplerenone treatment (AC3I‐No), and 15 wild‐type mice (WT‐Epler) receiving eplerenone treatment after TAC similar as the AC3I‐Epler group. Twelve‐week‐old mice of both sexes were used. All experiments were approved by the institutional ethical committee for animal experiments of the UMC Utrecht (DEC number 2013.II.11.115), and all procedures are performed conform the guidelines from Directive 2010/63/EU of the European Parliament on the protection of animals used for scientific purposes.

### 2.2 Experimental set‐up

All mice underwent TAC surgery. Procedures were conducted as previously described.^18^, ^19^ Mice were anesthetized by isoflurane (1.5%, in oxygen), intubated with a polyethylene catheter and ventilated with a rodent ventilator (Minivent, Hugo Sachs Electronics, Germany). A small incision in the second intercostal space was used to reach the aorta. Constriction was performed by tying a silk suture around the aorta together with a 27‐gauge needle, and then, the needle was subsequently removed. This latter procedure never took more than 12 seconds. Effective constriction was confirmed by Doppler echocardiography (see pressure gradient per group in Table 1). One week after TAC surgery, eplerenone treatment was started. Eplerenone tablets were pulverized and mixed with pulverized food, the food was weighed every week, and subsequent doses were calculated based on intake. After two, six and 12 weeks, all mice were again anesthetized by isoflurane and underwent cardiac echography. At 12 weeks, subsequent measurements were performed, consisting of electrocardiogram (ECG), epicardial mapping ex vivo, molecular biology and histology in vitro.

**TABLE 1:** Animal and functional characteristics at week 12

	AC3I‐No (n)	AC3I‐Epler (n)	WT‐Epler (n)
*Animal characteristics*			
Male	6 (16)	5 (14)	11 (15)
Bodyweight (g)	26,5 ± 1.0 (16)	25.5 ± 1.0 (14)	26.8 ± 0.8 (15)
Heart weight (g)	0.217 ± 0.016 (16)	0.189 ± 0.011 (14)	0.229 ± 0.014 (15)
HW/BW (mg/g)	8.1 ± 0.5 (16)	7.4 ± 0.2 (14)	8.6 ± 0.5 (15)
Tibia Length (cm)	1.81 ± 0.01 (16)	1.80 ± 0.01 (14)	1.80 ± 0.01 (15)
HW/TL (mg/cm)	119.8 ± 8.7 (16)	105.2 ± 8.2 (14)	128.1 ± 8.1 (15)
Lungs weight (g)	0.223 ± 0.029 (16)	0.164 ± 0.009 (14)	0.181 ± 0.02 (15)
Liver weight (g)	1.09 ± 0.05 (16)	1.10 ± 0.06 (14)	1.13 ± 0.05 (15)
Kidney weight (g)	0.157 ± 0.007 (16)	0.147 ± 0.006 (14)	0.145 ± 0.06 (15)
Average daily dose Eplerenone (mg/kg)	‐	207. 5 ± 8.1 (14)	190.6 ± 11.4 (15)
*Functional characteristics*			
RR (ms)	107.3 ± 4.7 (16)	110.4 ± 3.9 (14)	114.0 ± 5.0 (14)
PR (ms)	46.1 ± 1.5 (16)*	45.8 ± 1.6 (14)*	39.9 ± 0.9 (14)
P (ms)	11.2 ± 0.4 (16)	10.7 ± 0.5 (14)	10.0 ± 0.5 (14)
ORS (ms)	10. 4 ± 0.4 (16)	9.9 ± 0.5 (14)	10.6 ± 0.5 (14)
QTc (ms)	45.0 ± 4.1 (16)	48.8 ± 3.3 (14)	55.1 ± 2.1 (14)
Pressure gradient (mm Hg)	53.43 ± 3.07 (16)	56.39 ± 3.89 (14)	62.20 ± 4.55 (15)
CO (mL/min)	16.1 ± 0.92 (16)	15.42 ± 0.81 (14)	17.2 ± 1.48 (15)
EF (%)	45.23 ± 3.4 (16)	50.69 ± 3.65 (14)*	39.08 ± 2.54 (15)
FS (%)	22.52 ± 1.97 (16)	25.77 ± 2.14 (14)*	18.99 ± 1.33 (15)
LVESV (μL)	72.15 ± 9.32 (16)	65.73 ± 7.57 (14)	78.01 ± 6.09 (15)
LVEDV (μL)	96.99 ± 8.76 (16)	95.68 ± 7.28 (14)	99.94 ± 6.19 (15)
LVAW;d (mm)	1.23 ± 0.05 (16)	1.23 ± 0.05 (14)	1.19 ± 0.06 (15)
LVAW;s (mm)	1.54 ± 0.05 (16)	1.55 ± 0.05 (14)	1.44 ± 0.08 (15)
LVPW;d (mm)	1.40 ± 0.07 (16)	1.43 ± 0.06 (14)*	1.21 ± 0.07 (15)
LVPW;s (mm)	1.54 ± 0.07 (16)	1.67 ± 0.06 (14)*	1.41 ± 0.07 (15)
LVID;d (mm)	3.9 ± 0.10 (16)	3.62 ± 0.10 (14)*	4.23 ± 0.09 (15)
LVID;s (mm)	3.28 ± 0.14 (16)	2.84 ± 0.12 (14)*	3.55 ± 0.12 (14)
GLS (%)	‐12.11 ± 0.95 (15)	‐15.02 ± 1.24 (14)	‐11.66 ± 0.73 (15)
Average peak (%)	‐11.31 ± 0.9 (15)	‐13.74 ± 1.14 (14)	‐9.55 ± 0.93 (15)
Average time to peak (ms)	60.47 ± 3.63 (15)	66.57 ± 2.17 (14)	65.27 ± 2.86 (15)
Segmental dyssynchrony (ms)	11.49 ± 1.2 (14)	10.14 ± 0.73 (14)*	14.02 ± 1.53 (15)
Arrhythmia inducibility (%)	21 (14)	7 (14)	15 (13)

Note

All values are ± SEM; Standard error of the mean.

Abbreviations: Arrhythmia inducibility: ventricular fibrillation or ventricular tachycardia upon programmed electrical stimulation; CO: cardiac output; EF: ejection fraction; FS: fractional shortening; GLS: global longitudinal strain; HW/BW: heart weight corrected for bodyweight; HW/TL: heart weight corrected for tibia length; LVAW;d,s: left ventricular anterior wall thickness in diastole and systole, respectively; LVESV/LVEDV: LV end‐systolic/diastolic volume in μL; LVID;d,s: left ventricular internal dimmmhg

ensions diastole and systole, respectively; LVPW:d,s: left ventricular posterior wall thickness in diastole and systole, respectively.

* One‐way ANOVA, *P* < 0.05 compared to WT‐Epler.

### 2.3 Electrocardiography and echocardiography

Echocardiography was performed to determine functional and structural characteristics (VisualSonics, Vevo 2100 Imaging System). Echocardiographic data collected at two, six and twelve weeks were analysed using conventional analysis techniques in Vevo ImageLab. Data collected at two and twelve weeks were additionally analysed with the Vevo ImageLab Strain analysis software package. Long axis views were used for assessment of longitudinal strain and time to peak (T2P). The endocardial border was traced and divided in 48 individual points, eight points together composed one endocardial segment which provided six endocardial segments. Strain is defined as change in length between relaxation and contraction. Strain dyssynchrony is evaluated as being the standard deviation (STDEV) of the time to peak (T2P) of the six different endocardial segments per mouse. The higher the STDEV T2P per mouse, the more dyssynchrony is present.^20^

Prior to sacrifice at 12 weeks, in anesthetized mice (1.5% isoflurane), a 3 lead ECG was recorded on a custom‐built ECG‐amplifier and analysed offline using Chart 5 Pro (AD Instruments, Dunedin, New‐Zealand).

### 2.4 Langendorff perfusion and epicardial mapping

After in vivo measurements, the heart was excised and connected to a Langendorff perfusion set‐up. Perfusion buffer contained (mmol/L) NaCl 116, KCl 5, MgSO_4_ 1.1, NaH_2_PO_4_ 0.35, Na HCO_3_ 27, glucose 10, mannitol 16 and CaCl_2_ 1.8, which was carbogen‐gassed and kept at 37ºC. A multi‐electrode 16×13 grid was placed on the epicardial surface of the heart. Stimulation was performed from the centre of the grid (2× stimulation threshold). Assessment of arrhythmias was performed in three steps, (a) spontaneous arrhythmias, (b) arrhythmias induced by a 16‐paced train (BCL 100ms) followed by 1‐3 premature stimuli close to the effective refractory period and (c) arrhythmias induced by 2‐seconds burst pacing with the shortest possible cycle length. Arrhythmias were classified as sustained when ventricular tachycardia or fibrillation (VT/VF) lasted for more than 15 consecutive beats.

### 2.5 Histology

Hearts were removed from the Langendorff apparatus, quickly frozen in liquid nitrogen and stored at −80ºC. Four‐chamber view cryo‐sections were generated for histochemistry to assess fibrosis using picrosirius red staining. For quantification, sections were digitally scanned using Aperio ScanScope software (Leica Microsystems BV, Son, The Netherlands) and pictures were taken using ImageScope software (Leica Microsystems BV). From each mouse heart, two or three slices were used for quantification of fibrosis using ImageJ 1.35s software. Fibrosis was calculated as the percentage of total ventricular area. For total fibrosis, no additional protocol was used. To discriminate between patchy fibrosis and interstitial fibrosis, a Gaussian blur (sigma = 2) was used, visualizing fibrosis in greyscale. This greyscale corresponded with the type of fibrosis, where black was patchy fibrosis and light grey was interstitial fibrosis.

### 2.6 Real‐time quantitative PCR

From pulverized cardiac tissue, total RNA was isolated using TRIzol reagent (Invitrogen, Carlsbad, CA, USA) and subsequently treated with DNAse I. Two μg DNAse treated RNA was used to convert into cDNA with Reverse Transcriptase (Invitrogen) according to the manufacturer's protocol. Obtained cDNA was diluted ten times prior to use. RT‐qPCR was performed using TaqMan Gene Expression Assays (Applied Biosystems by Life Technologies Corp., Carlsbad, CA, USA). Relative mRNA levels were determined of collagen 1α1 (*Col1α1*), collagen 1α2 (*Col1α2*), collagen 3α1 (*Col3α1*), transforming growth factor β (*Tgf‐β*) and nuclear factor κb (*NF‐κB*). The geometric mean of *Gapdh* and TATA‐binding protein (*Tbp*) was used as internal control. AC3I‐No and WT‐Epler groups are shown as relative fold increase compared to the AC3I Epler group.

### 2.7 Immunoblotting

Total protein was isolated from cardiac tissue using radioimmunoprecipitation buffer (10 mmol/L Tris‐HCL, 150 mmol/L NaCl, 1% triton X‐100, 0.1% sodium dodecyl sulphate (SDS), and 0.5% sodium deoxycholate, pH 7.4). Total protein was separated on a 7% or 10% SDS‐Page gel and transferred to a nitrocellulose membrane. Membranes were blocked for one hour using either 5% milk powder or 5% bovine serum albumin (BSA) dissolved in TBST depending on the primary antibody used. Membranes were incubated overnight at 4°C with a mouse monoclonal antibody against total SMAD 2/3 (1:200, Santa Cruz, Inc., Dallas, TX, USA), smooth muscle actin (SMA, 1:500, Sigma, St. Louis, MO, USA), a rabbit monoclonal antibody against phosphorylated SMAD3 (pSMAD3, 1:1000, Abcam, Cambridge, UK), a rabbit polyclonal antibody against fibronectin (1:1000, Sigma‐Aldrich, St. Louis, MO, USA), vimentin (1:1000, Thermo Scientific, Waltham, MA, USA) or phosphorylated (at Thr^17^) phospholamban (pPLN, 1:250 Santa Cruz, Inc). Primary antibodies were dissolved in 5% milk powder or 5% BSA dissolved in TBST. Detection of the primary antibody was achieved by one‐hour incubation at 4°C with a secondary antibody directed against mouse or rabbit IgG (1:250 in 5% milk powder or BSA in TBST) and was visualized by an ECL detection kit (GE healthcare, Chicago, IL, USA). Bands were detected on a ChemiDoc XRS+ (Bio‐Rad, Hercules, CA, USA) and ImageLab (version 6.0.0, Bio‐Rad).

### 2.8 Statistical analysis

Data were showed as means ± standard error of the mean (SEM). Statistics were performed by one‐way ANOVA with a post hoc test (Tukey's HSD) or Student's *t* test when appropriate. Differences were considered significant if *P* < 0.05. All analyses were performed using GraphPad Prism 6.0 (GraphPad Software, La Jolla, CA, USA).

## 3 RESULTS

### 3.1 Animal characteristics and AC3I effectivity—model validity

There was no difference in bodyweight, heart weight, heart weight corrected for bodyweight or tibia length, or other organ weights at 12 weeks post‐intervention (Table 1). Furthermore, there was no difference in daily intake of eplerenone between the AC3I‐Epler and WT‐Epler group (Table 1). In the presence of the AC3I peptide, phosphorylation of phospholamban (pPLN) at Thr^17^ was significantly decreased indicating an active and potent CaMKII inhibition in our AC3I mice (Figure S1).

### 3.2 Conventional echocardiographic measurements

**FIGURE 1:**
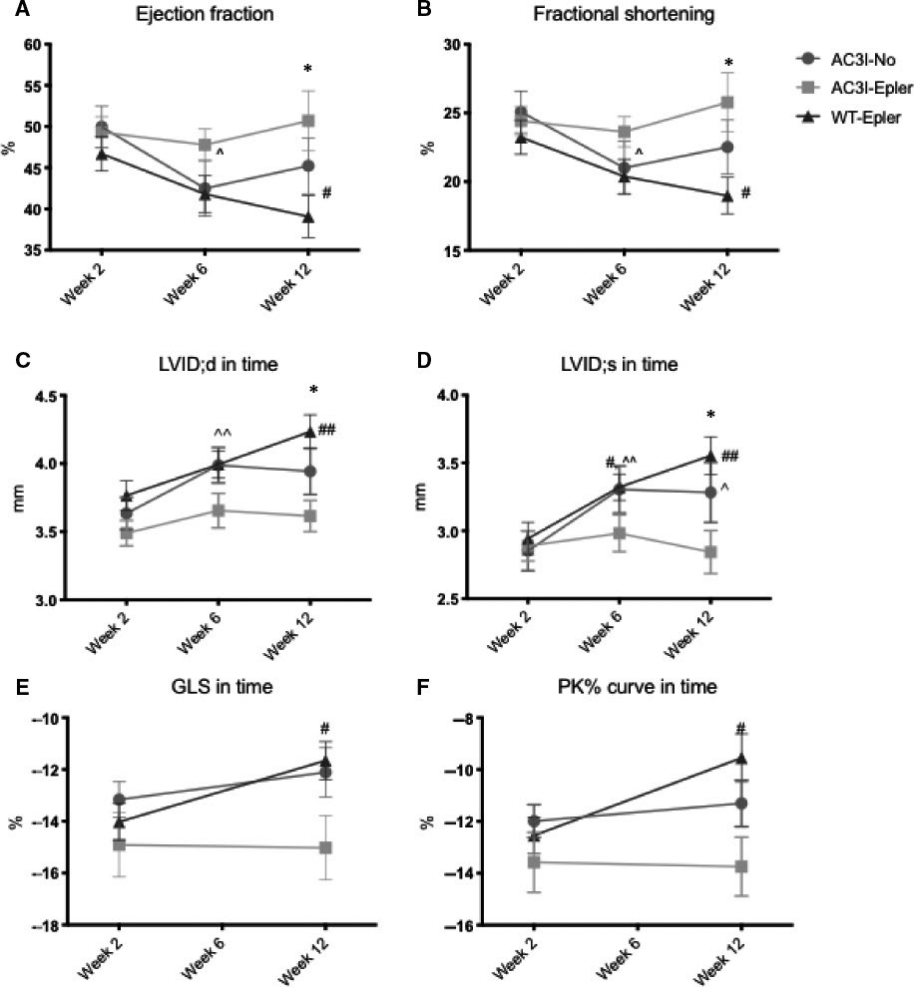
AC3I‐Epler mice showed a significant better ejection fraction (A) and fractional shortening (B) compared to WT‐Epler mice at twelve weeks of TAC. (C,D) In diastole, AC3I‐No mice showed a significantly dilated LV compared to two weeks, while WT‐Epler mice show a significantly dilated LV at twelve weeks compared to 2 weeks. E, A decrease in global longitudinal strain (GLS) and, (F) average peak % of WT‐Epler mice in time, where AC3I‐Epler mice preserved a constant GLS and PK%, while AC3I‐No animals showed a small but insignificant reduction in GLS and PK% in time. EF and FS at weeks 2, 6 and 12: AC3I‐No n = 16, AC3I‐Epler n = 14, WT‐Epler n = 15. GLS and PK% at week 2 and 12: AC3I‐No n = 15, AC3I‐Epler n = 14, WT‐Epler n = 15. One‐way ANOVA or Student's *t* test when appropriate, *P < 0.05 AC3I‐Epler compared to WT at specific time point. ^#^ *P* < 0.05 compared to WT week 2, ^^^ *P* < 0.05 compared AC3I‐No week 2. ^##^ *P* < 0.01 compared to WT‐Epler week 2, ^^^^ *P* < 0.01 compared to AC3I‐No. All values are mean ± SEM

Echocardiography at 12 weeks showed no difference in pressure gradient across the aortic constriction, meaning that constrictions were comparable among groups. (all echo data listed in Table 1). Cardiac output (CO) proved to be equal between groups. However, ejection fraction (EF) was significantly higher in AC3I‐Epler animals (50.69 ± 3.65%) compared to WT‐Epler animals (39.08 ± 2.54%) suggesting a beneficial effect of AC3I and eplerenone treatment together. Chronic CaMKII inhibition alone increased the EF slightly in comparison with WT‐Epler, however, not significantly (45.23 ± 3.4%). The same holds true for fractional shortening (FS) where AC3I‐Epler mice performed significantly better than WT‐Epler animals (25.77 ± 2.14% vs 19.88 ± 1.33%, respectively). AC3I‐No animals performed at an intermediate level (22.52% ± 1.97). Measurements in time show that animals from all three groups had an equal EF and FS at week two (Figure 1A,B, respectively). However, WT‐Epler animals started to deteriorate both with regard to EF and FS at six weeks after TAC, which progressively further deteriorated to a significantly worse EF and FS compared to the time point of two weeks. AC3I‐No animals showed a decreased EF and FS at week six, and however, they seemed to recover slightly at 12 weeks. AC3I‐Epler animals did not deteriorate at all, neither at 6 weeks nor at twelve weeks post‐TAC, and displayed even a small increase in EF and FS at 12 weeks, although this was not significant (Table 1, Figure 1A,B). With regard to structural remodelling, LV anterior wall thickness in diastole and systole (LVAW;d and LVAW;s, respectively) did not differ between groups, and however, there was a significant increase in posterior wall thickness in both diastole and systole in AC3‐Epler animals when compared to WT‐Epler animals at twelve weeks (LVPW;d 1.43 ± 0.06 vs 1.21 ± 0.07, LVPW;s 1.67 ± 0.06 vs 1.41 ± 0.07, respectively, Table 1). Analysis of LV internal dimensions in diastole and systole revealed a significant LV dilation over time in both AC3‐No and WT‐Epler animals, whereas LV dimensions remained constant in AC3I‐Epler animals (Figure 1C,D). This resulted in a significantly more dilated status in WT‐Epler mice compared to AC3I‐Epler animals both in diastole and systole at twelve weeks (LVID;d 4.23 ± 0.10 mm vs 3.66 ± 0.10 mm, LVID;s 3.56 ± 0.12 mm vs 2.84 ± 0.12 mm, respectively, Table 1).

### 3.3 Echocardiographic strain analysis

Global longitudinal strain (GLS) is defined as the fractional change in total length of the U‐shaped endocardial contour. The GLS at 2 weeks after TAC surgery was moderately reduced (normal GLS is approximately −18)^21^ but comparable among groups (AC3‐No −13.16 ± 0.68%; AC3I‐Epler −14.91 ± 1.24%; WT‐Epler −14.02 ± 0.71%) as is shown in Figure 1E. Although at twelve weeks no differences between groups were found, the WT‐Epler mice did show a significantly deteriorated GLS (−11.66 ± 0.73%) compared to two weeks (14.02 ± 0.71%, *P* < 0.05, Table 1 and Figure 1E). AC3I‐No and ACI‐Epler animals showed preserved GLS at twelve weeks compared to two weeks (−12.11 ± 0.95% vs −13.16 ± 0.68% and −15.02 ± 1.25% vs −14.91 ± 0.71%, respectively, Table 1 and Figure 1E). The same accounts for the average peak % (Table 1 and Figure 1F), which is an average of the maximum deformation in percentage of the six endocardial segments measured (see Figure S2A for an example of a strain analysis graph, the grey line with the black dot depicts the average curve). At two weeks, peak % was equal among the groups (AC3I‐No −11.99 ± 0.63%, AC3I‐Epler −13.58 ± 1.16%, WT‐Epler −12.54 ± 0.7%). At 12 weeks, AC3I‐No and AC3I‐Epler mice had a preserved average peak % (−11.31 ± 0.9% and −13.74 ± 1.14%, respectively), and however, the WT‐Epler mice had a significantly decreased average peak % (−9.55 ± 0.93% *P* < 0.05) when compared to the peak % at 2 weeks (Table 1 and Figure 1F).

**FIGURE 2:**
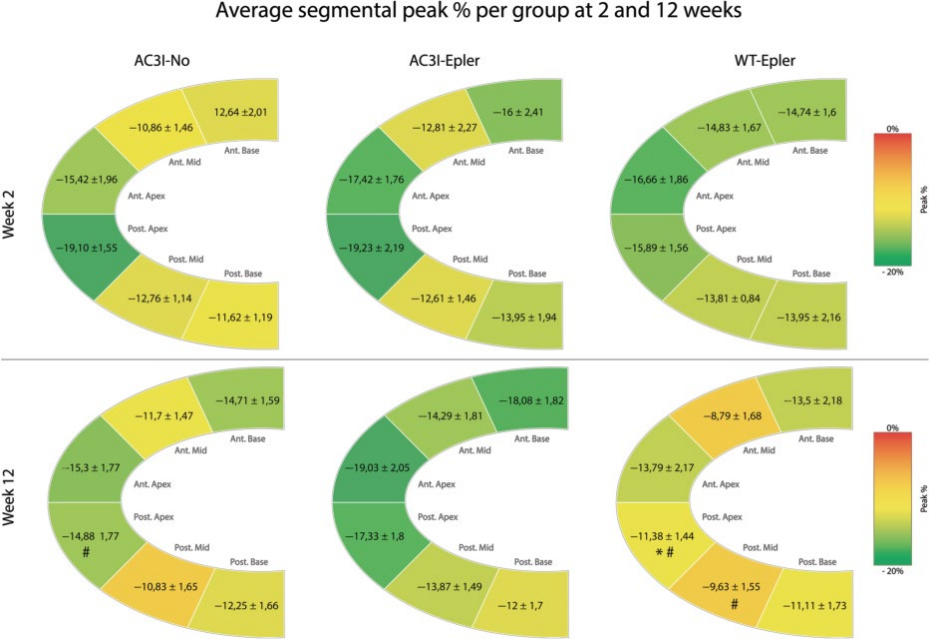
Figure depicts the murine myocardium divided in six segments. Per segment, the peak strain in % is reported with mean values ± SEM. WT‐Epler mice have a decreased peak strain % at 12 wk compared to 2 wk which is mostly because of significant worsening of strain in the posterior middle and apical segments. One‐way AVOVA, *P < 0.05 compared to AC3I‐Epler at specific time point. Student's *t* test, ^#^ *P* < 0.05 compared corresponding segment in week 2. Week 2: AC3I‐No n = 16, AC3I‐Epler n = 14, WT‐Epler n = 14, week 12: AC3I‐No n = 15, AC3I‐Epler n = 14, WT‐Epler n = 15

Focusing on peak % per segment, in week two we observed comparable values between segments in each group and comparable percentages between groups. This is indicated as a reasonably homogenous colour in the schematic endocardial diagram shown in Figure 2. At 12 weeks, we observed in the AC3I‐Epler animals a preserved strain expressed as peak % in all six segments, hence the same tincture in the diagram in Figure 2. The AC3I‐No animals showed a preserved strain in all segments at 12 weeks except for the posterior middle segment were the peak strain significantly decreased from −19.1 ± 1.55% to −14.88 ± 1.77%, and this is visualized as a more yellow colour at twelve weeks compared to the corresponding segment at two weeks in Figure 2. WT‐Epler mice showed a significantly decreased average peak % at twelve weeks compared to two weeks (Figure 1F), and this is predominantly caused by deterioration of strain at the posterior middle (−13.81 ± 0.84% vs 9.63 ± 1.55% *P* < 0.05) and posterior apical segments (−15.89 ± 1.56% vs −11.38 ± 1.44% *P* < 0.05) as is depicted in Figure 2. Also, when we compared the three groups with each other at 12 weeks, the posterior apical segment of the WT‐Epler animals performed significantly worse compared to the posterior apical segment of the AC3I‐Epler animals (−17.33 ± 1.8% *P* < 0.05).

**FIGURE 3:**
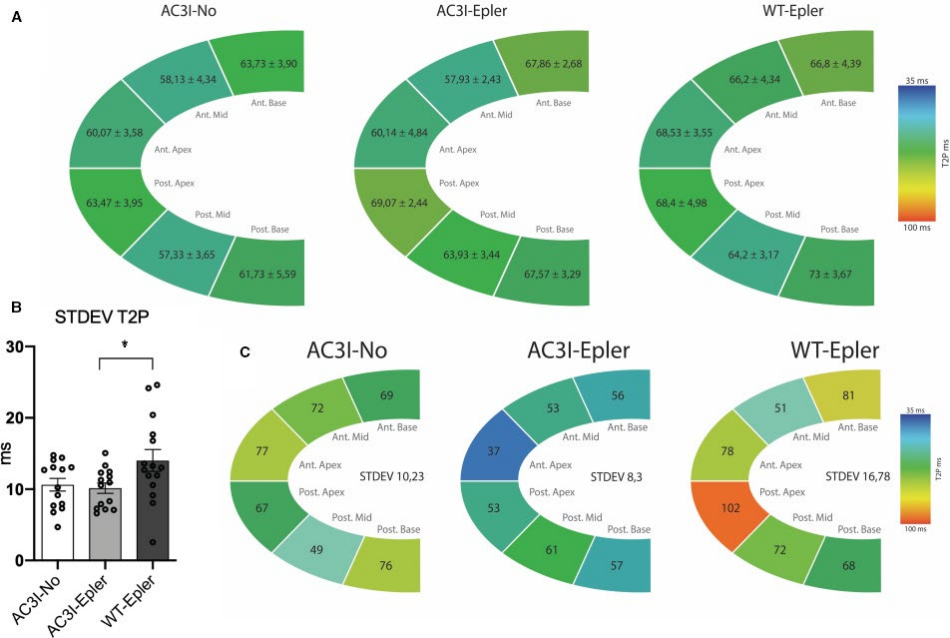
Figure depicts the murine myocardium divided in six segments. Per segment, the time to peak (T2P) in ms is reported. All values are mean ± SEM. A, Mean values per group at week 12 are depicted. No differences in T2P of the different segments between groups are observed. B, Within each individual mouse, the standard deviation (STDEV) of the T2P of the six segments was calculated to evaluate dyssynchrony, in the graph the mean STDEV per group is reported at 12 wk. WT‐Epler mice experienced a significant higher level of dyssynchrony compared to AC3I‐Epler mice. C, Representative examples of T2P per group, the more heterogeneous the colour pattern the more dyssynchrony is present. AC3I‐No n = 15, AC3I‐Epler n = 14, WT‐Epler n = 15. One‐way ANOVA, *P < 0.05

In Figure 3A, we show per group the average time it takes for each segment to reach its maximal peak strain, T2P, at 12 weeks. Depicted data show homogenous green diagrams indicating no notable delay of one of the segments in either of the groups. To evaluate dyssynchrony, we calculated the STDEV of the T2P of the six segments per mouse since the higher the STDEV T2P, the more dyssynchrony is present. Examples of dyssynchronous strain analysis are shown in Figure S2B and 2C. AC3I‐No animals showed a STDEV T2P of 11.49 ± 1.2 ms, which was comparable to the STDEV T2P of the AC3I‐Epler mice of 10.14 ± 0.73 ms (Figure 3B). However, the WT‐Epler mice showed significantly more dyssynchrony (14.02 ± 1.53 ms, Figure 3B) compared to AC3I‐Epler animals. Segmental diagrams of representative mice are shown in Figure 3C, the diagrams of the AC3I‐Epler and AC3I‐No animals are reasonably homogenous in colour, whereas the diagram of the WT‐Epler animal is strikingly more variable.

### 3.4 Electrocardiogram parameters and arrhythmia inducibility

Before sacrificing the mice, an ECG was recorded. No differences were observed in ECG parameters with exception of the PR interval which was significantly shorter in WT‐Epler mice compared to AC3I‐No and AC3I‐Epler mice (Table 1). Arrhythmia inducibility was tempered in the AC3I‐Epler group, but not significantly suppressed; AC3I‐No 21% vs AC3I‐Epler 7% vs WT‐Epler 15%. No significant differences in morphology or duration of the arrhythmias could be observed due to the general low incidence of arrhythmias.

### 3.5 Degree of Interstitial and patchy fibrosis

**FIGURE 4:**
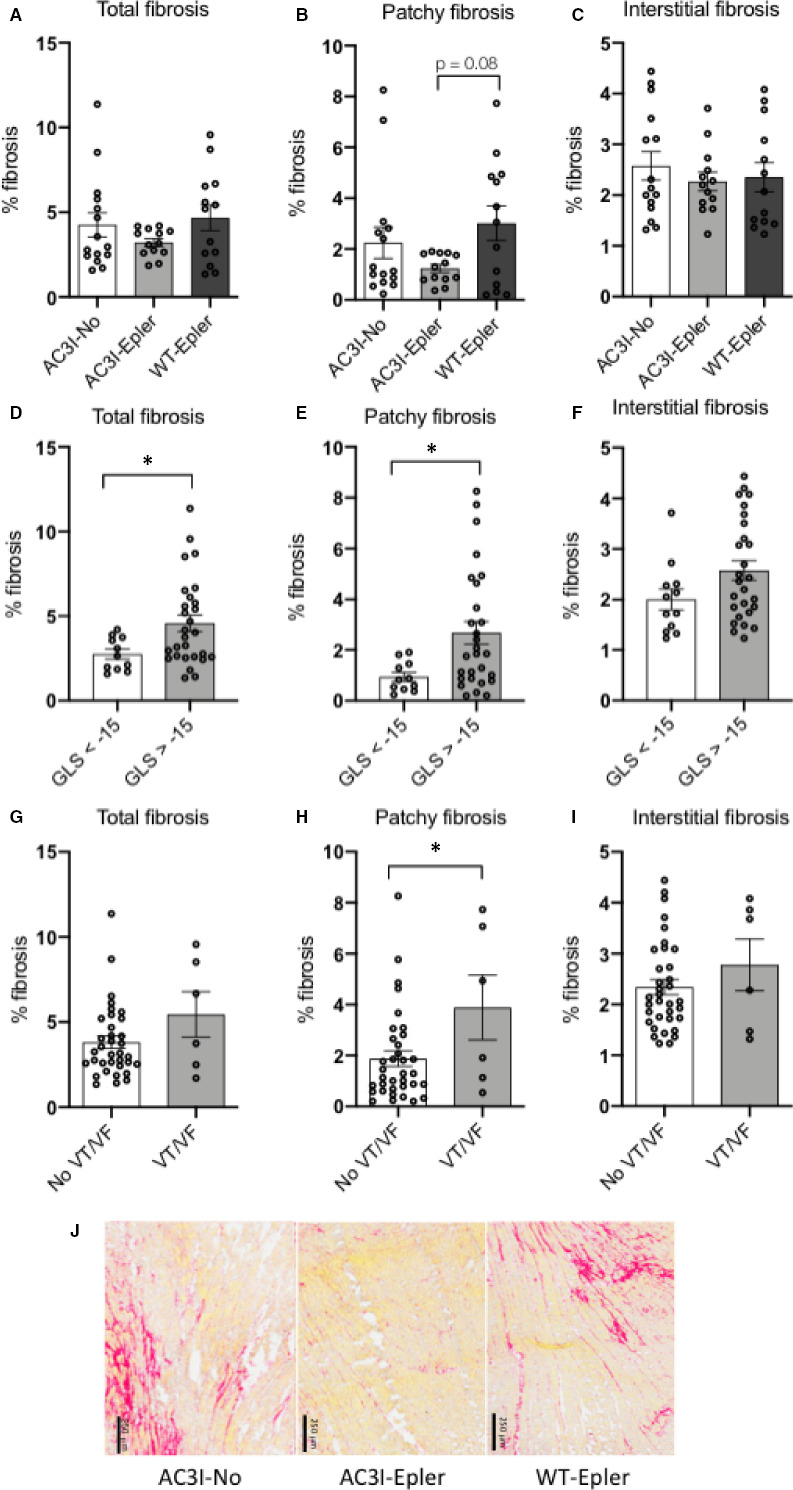
Figure depicts fibrosis levels at twelve weeks. The subtle decrease in total fibrosis (A) in AC3I‐Epler mice is mainly caused by a trend towards reduced patchy fibrosis (B, patchy fibrosis AC3I‐Epler vs WT‐Epler *P* = 0.08), where interstitial fibrosis in all groups is equal (C). (D, E and F) show all mice divided in two groups, animals with a moderate or good GLS (more negative than ‐ 15), or a bad GLS (less negative than ‐ 15). (D and E) illustrate a lower percentage of total and patchy fibrosis in mice with a moderate to good GLS. F, shows that there is no difference in interstitial fibrosis between mice with a bad GLS. G, No significant difference in total fibrosis and interstitial fibrosis when mice which do not show inducible arrhythmias are compared to mice which do show inducible arrhythmias (I). H, The difference in patchy fibrosis is significant between inducible and non‐inducible mice. J, Representative pictures of heart slices stained using Sirius Red focusing on patchy fibrosis. In AC3I‐no and WT‐Epler mice, there was more patchy fibrosis present compared to AC3I‐Epler mice. Interstitial fibrosis was equal among groups. For n per group, see Table S1. A‐C one‐way ANOVA, D‐I Student's *t* test, *P < 0.05. Graphs depict means ± SEM. GLS: global longitudinal strain. VT/VF: ventricular tachycardia/ fibrillation

**FIGURE 5:**
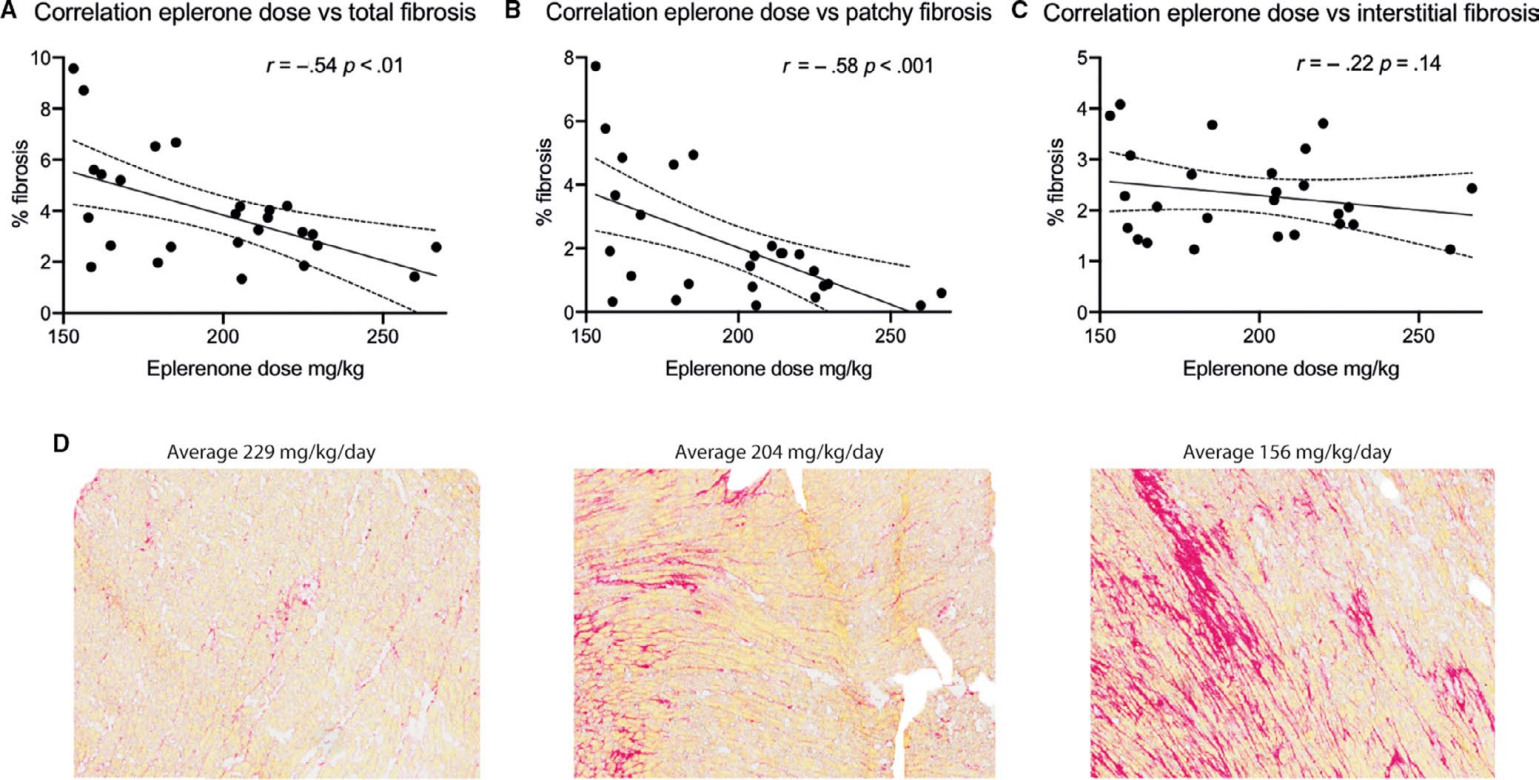
Figure shows correlations between fibrosis at twelve weeks (in %) and average eplerenone doses per day (intake over 11 wk regardless of genotype). A, Significant correlation between eplerenone dose and total fibrosis, which is mainly determined by, the correlation between eplerenone dose and patchy fibrosis (B). C, No correlation was found between eplerenone dose and interstitial fibrosis. D, Examples of fibrosis deposition with their corresponding eplerenone doses. Vessels and perivascular fibrosis were removed before analysis. A‐C Pearson's correlation coefficient was calculated, n = 29

Total tissue fibrosis levels proved to be equal among groups; AC3I‐No 4.26 ± 0.72%, AC3I‐Epler 3.22 ± 0.23% and WT‐Epler 4.67 ± 0.76% as is shown in Figure 4A. However, when we discriminated between interstitial fibrosis and patchy fibrosis, we observed slight differences in patchy fibrosis (Figure 4B), and absolutely no variation in interstitial fibrosis between our three study groups (Figure 4C). The lower percentage of patchy fibrosis in AC3I‐Epler mice showed a trend towards reduced patchy fibrosis in the AC3I‐Epler mice (*P* = 0.08) when compared to WT‐Epler mice (representative images shown in Figure 4J). When we divided our mice according to GLS at twelve weeks as being better (<), or worse (>) than −15 (−15 as being moderate GLS), we noticed that mice with a less negative GLS had significantly more total (4.58 ± 2.60% vs 2.75 ± 0.97%, Figure 4D) and patchy (2.67 ± 2.35% vs 0.94 ± 0.59%, Figure 4E, Table S1) fibrosis. Interstitial fibrosis was comparable among groups (Figure 4F), suggesting that patchy fibrosis could be an important factor affecting function. Comparing fibrosis in regard of arrhythmia inducibility, we noticed a trend towards a higher amount of total fibrosis in animals which showed inducible arrhythmias (5.45 ± 1.34%) compared to animals without inducible arrhythmias (3.81 ± 0.36%, Figure 4G). In animals with inducible arrhythmias, there was a significant higher amount of patchy fibrosis (3.89 ± 1.27%, Figure 4H) compared to animals that were not inducible (1.87 ± 0.3%). Furthermore, the level of interstitial fibrosis did not differ between inducible and not inducible mice (2.78 ± 0.51% vs 2.34 ± 0.15%, respectively, Figure 4I), again indicating that patchy fibrosis plays a role in arrhythmogenicity. Interestingly, we found a significant correlation between average eplerenone dose per day (intake during eleven weeks) and total fibrosis at week twelve (n = 26, *r* = −0.54, *P* < 0.01, Figure 5A). This correlation was also present, and slightly more pronounced, between eplerenone dose and patchy fibrosis (n = 26, *r* = −0.58, *P* < 0.001 Figure 5B), but absent between eplerenone dose and interstitial fibrosis (n = 26, *r* = −0.22, *P* = 0.14, Figure 5C). Examples of fibrosis deposition with their corresponding eplerenone dose are depicted in Figure 5D. This correlation indicates that eplerenone primarily tempers patchy fibrosis deposition but does not affect interstitial fibrosis. Fibrosis data are summarized in Table S1.

### 3.6 Pro‐fibrotic signalling pathways

The mRNA expression of *Tgf‐β* and *Nf‐κb* was determined. *Tgf‐β* mRNA was lowest in AC3I‐Epler, with a modest increase in expression in AC3I‐No and a significant increase in expression in WT‐Epler (Figure S3A). Furthermore, we detected a significant higher expression of *Nf‐κb* mRNA in WT‐Epler mice (Figure S3B). NF‐κB is involved in pro‐inflammatory and pro‐remodelling pathways.

Following up on the difference in *Tgf‐β* mRNA, we investigated the underlying SMAD3 pathway. Total SMAD3 protein was equally present in all three groups (Figure S3C,D), whereas a significant decrease in phosphorylated SMAD3 was observed in both the AC3I‐No as in AC3I‐Epler mice (Figure S3E,F). When looking at downstream targets of the SMAD3 pathway in pooled protein (n = 5) samples, we noticed a confirming pattern. Fibronectin, vimentin and α‐smooth muscle actin (α‐SMA) expression was highest in WT‐Epler mice, and lower in both AC3I groups as is shown in Figure S3G‐J. This suggests that CaMKII inhibition displays an inactivating effect on the TGF‐β/SMAD3 signalling pathway.

## 4 DISCUSSION

Recently, we showed that in mice subjected to pressure overload, chronic CaMKII inhibition: (a) preserved conduction velocity, expression and localization of the gap junction protein Cx43 and Nav1.5 sodium channels, (b) did not prevent hypertrophy and fibrosis formation and consequently and (c) did not prevent heart failure and arrhythmogenesis^7^). As a follow‐up, we hypothesized that under these conditions, chronic administration of the MR antagonist eplerenone would mitigate deterioration of cardiac function. This because in a previous study in aged mice, chronic eplerenone treatment not only limited (especially patchy) fibrosis formation, but as a consequence also limited arrhythmogenesis.^14^ Beyond the consequences for cardiac contractile performance, we were also interested to explore the effect of the intervention on arrhythmogenesis. The latter because fibrosis formation not only gives rise to inhomogeneity of contraction but also triggers conduction disturbance, which additionally is depending on CaMKII activity. Data obtained in this current study showed that in mice with chronic pressure overload, a combined CaMKII inhibition together with MR antagonism: (a) mitigates contractile deterioration as was manifested by a preservation of EF, FS, GLS, peak strain and contractile synchronicity during the 12 weeks of pressure overload. (b) reduces patchy fibrosis formation, potentially via inhibition of pro‐fibrotic TGF‐β/SMAD3 signalling, which again related to a preservation of function and slightly depressed incidence of arrhythmias.

### 4.1 Conceptual framework

**FIGURE 6:**
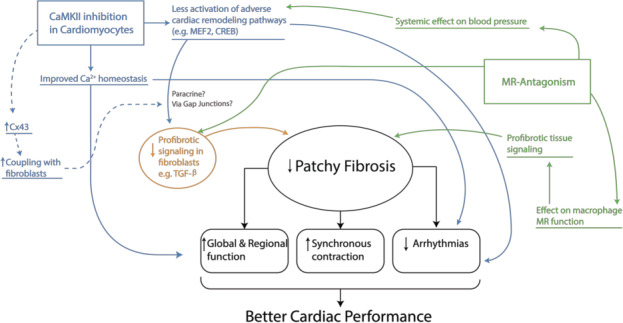
Schematic overview of the different pathways affected by CaMKII inhibition and mineralocorticoid receptor antagonism in this study. For detailed description, see Discussion

The effects of CaMKII on intracellular Ca^2+^ handling have direct consequences for cardiac function (Figure 6). CaMKII mediated phosphorylation of the ryanodine receptor increases the open probability, phosphorylation of the L‐Type Ca^2+^ channel leads to a slower inactivation, and phosphorylation of phospholamban leads to an increase in sarcoplasmic Ca^2+^ load.^22^, ^23^ Resulting from these actions, up‐regulated CaMKII activity during cardiac pathology leads to an increased intracellular Ca^2+^ concentration and can lead to triggered activity via spontaneous diastolic Ca^2+^ release.^9^, ^24^

Next to its effects on calcium handling, CaMKII also exerts its effects on cardiac function via regulation of gene expression, as is also depicted in Figure 6. Directly via phosphorylation of transcription factors, or indirectly by phosphorylation of transcription factor modulators.^4^, ^25^ CaMKII is known to directly phosphorylate CREB (cAMP response element‐binding protein) and SRF (serum response factor) transcription factors, which probably have a role in transcription of genes involved in cardiac dilatation, contractile performance and preservation of intercellular integrity.^26^, ^27^ CaMKII indirectly induces activation of the MEF2 (myocyte enhancer factor) transcription factor via phosphorylation of HDAC4 (histone deacetylase) which results in translocation of HDAC4 to the cytoplasm, alleviating repression of MEF2.^28^ MEF2 activates a gene programme resulting in loss of sarcomere structure and subsequent contractile dysfunction.^29^, ^30^, ^31^ Furthermore, it is known that CaMKII indirectly regulates *NF‐κB* (nuclear factor κB) via IκB leading to intranuclear translocation of *NF‐κB*. In the nucleus, *NF‐κB* regulates a gene programme involved in adverse remodelling and inflammation.^32^, ^33^ In our current study, inhibition of CaMKII with the AC3I peptide in our mouse model results in tempering of these pro‐remodelling pathways which is in line with the observed effects on the expression of *Tgf‐β* and *Nf‐κb* mRNA, and fits with the improved contractile performance of these animals under conditions of chronic pressure overload.

CaMKII inhibition leads to increased intercellular coupling via Cx43 gap junctions, as we showed previously.^7^ In models of cardiac hypertrophy, it is commonly observed that an increase in collagen deposition is preceded by reduction in Cx43 and that aligns with increased fibroblast activity, although the exact underlying mechanism is unknown.^19^, ^34^ It is known that cardiomyocytes and fibroblasts are able to form functional gap junctions via Cx43 hemichannels,^35^ and therefore, direct communication via small molecules (<1 kD) between cardiomyocytes and fibroblasts can be more effective when increased expression of functional Cx43 channels is present. However, recent findings in skin fibroblasts suggest a direct role for Cx43 in regulation of gene expression since blocking Cx43 hemichannels resulted in a up‐regulation of pERK1/2 and a subsequent increase in *TGF‐β* mRNA.^36^, ^37^ Here, we show a decrease in *Tgf‐β* mRNA expression upon CaMKII inhibition which theoretically can be mediated via increased Cx43 coupling and a tempering of the pERK1/2 pathway. In neonatal rat fibroblasts, it was shown that collagen production is significantly controlled by the ERK1/2 pathway,^38^ and more studies suggest a pro‐fibrotic role of p‐ERK1/2 in cardiac fibroblasts.^39^, ^40^ This role potentially could be exerted via up‐regulation of the pro‐fibrotic TGF‐β/SMAD signalling cascade. Indeed, in our AC3I mice, the observed alleviation of the TGF‐β/SMAD signalling pathway fits with a preserved expression and functionality of Cx43 gap junction channels (Figure 6).^7^

Eplerenone treatment on top of CaMKII inhibition seems to potentiate the antifibrotic and anti‐arrhythmic effect, as can be concluded from the improved functional parameters and the effect on pro‐fibrotic signalling in our AC3I‐Epler mice. Normal MR signalling in cardiac tissue has several different types of target cells where it exerts it is pro‐fibrotic capacities. MR signalling in cardiomyocytes has been shown to induce CTGF expression,^41^ although the decisive role of CTGF in fibrosis formation is still subject of debate.^42^ Furthermore, MR signalling plays a role in the type of cardiac fibrosis that is formed. Inhibition of MR signalling in mice (via cardiomyocyte‐specific genetic deletion) inhibited reactive fibrosis and also reduced the expression of hypertrophy and fibrosis‐associated genes such as β‐myosin heavy chain, angiotensin‐converting enzyme, CTGF and collagens.^43^ The immune system also plays a significant role in the formation of cardiac fibrosis and also here MR signalling is involved (Figure 6). In this study, our mice were treated orally for 11 weeks with the selective mineralocorticoid receptor antagonist eplerenone, and thereby, they were subjected to a systemic inhibition of the different platforms of MR‐activity. For that, we cannot exclude the involvement of effects exerted on non‐cardiomyocytes. In endothelial cells, MR activation leads to increased expression of ICAM‐1 (intercellular adhesion molecule 1) which facilitates enhanced adhesion and subsequent infiltration of macrophages into the myocardium.^44^, ^45^ In macrophages, the MR plays a role in determining the macrophage response. MR activation in macrophages leads to a pro‐inflammatory M1, and a pro‐fibrotic M2 phenotype, with an increased TNFα and TGF‐β (and subsequent SMAD3 phosphorylation) production because of activation of the JNK/activator protein (AP)‐1 pathway.^46^, ^47^

### 4.2 Study limitations

The effect of chronic CaMKII inhibition via AC3I was not able to completely inhibit adverse remodelling in our model of pressure overload, but it should be noted that improvement of function is present while the trigger for remodelling (aortic constriction) persists. It would therefore not be realistic to expect a complete inhibition of remodelling.

## 5 CONCLUSION

The double treated AC3I‐Epler mice perform best in this study, which is most likely due to a combination of interference on all the above‐discussed actions of CaMKII in the cardiomyocyte and MR signalling in all cells present in the diseased myocardium. It is clear that the addition of eplerenone to CaMKII inhibition via AC3I does potentiate the effects of CaMKII inhibition on pro‐fibrotic pathways. As a result of the applied strategy, limiting patchy fibrosis adheres to a higher synchronicity of contraction and an overall better contractile performance which fits also with a slightly tempered arrhythmogenicity.

## CONFLICT OF INTEREST

None declared.

## AUTHOR CONTRIBUTIONS

Driessen HE, Fontes MS, and van Stuijvenberg L carried out the experiments. Driessen HE wrote the manuscript with support from van Veen TA and Vos MA. Brans M carried out the surgical procedures. Goumans MJ encouraged Driessen HE to investigate the fibrosis pathways. Van Veen TA supervised the project. All authors approved final version of the manuscript.

## Supporting information

Supplementary Material

## Data Availability

The data that support the findings of this study are available from the corresponding author upon reasonable request.
