# Supplementary material for: A combined CaMKII inhibition and mineralocorticoid receptor antagonism via eplerenone inhibits functional deterioration in chronic pressure overloaded mice
Source: J Cell Mol Med. 2020 Jun 23;24(15):8417–29. doi: 10.1111/jcmm.15355 (PMC7412412; doi:10.1111/jcmm.15355)
Supplement: Supplementary file 1 — Supplementary Material [file JCMM-24-8417-s001.docx]

# Supplemental Material

***Supplemental figure 1.***


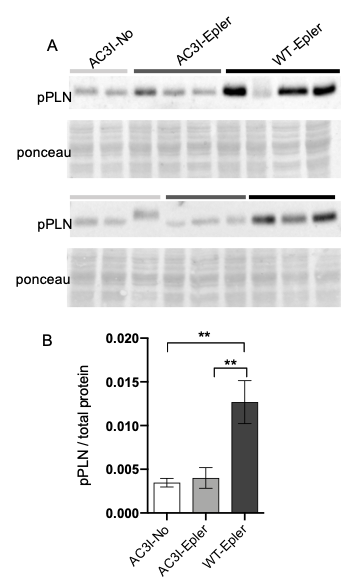


Western blot results (A) and quantification of pPLN (phosphorylated PLN, B) on mice from all three groups, AC3I-No n=5, AC3I-Epler n=6, WT-Epler n=7. Graphs show a significant reduction of pPLN in AC3I mice compared to WT mice showing effectivity of CaMKII inhibition on PLN phosphorylation. One-way ANOVA, ** p<0.01.

***Supplemental figure 2.***


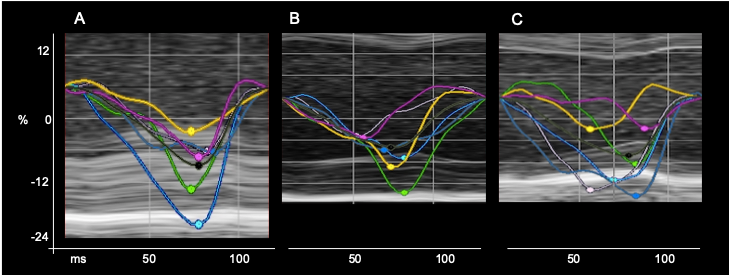


Examples of strain analysis graphs. Color of graph represents different endocardial segments. Green; posterior base. White; posterior middle. Light blue; posterior apex. Dark blue; anterior base. Yellow; anterior middle. Purple; anterior apex. Grey; average curve. Examples of (A) synchronous segmental strain, with moderately reduced peak strain. (B) Moderate dyssynchronous segmental strain with severe reduced peak strain. (C) Severe dyssynchronous segmental strain and reduced peak strain.

***Supplemental figure 3.***


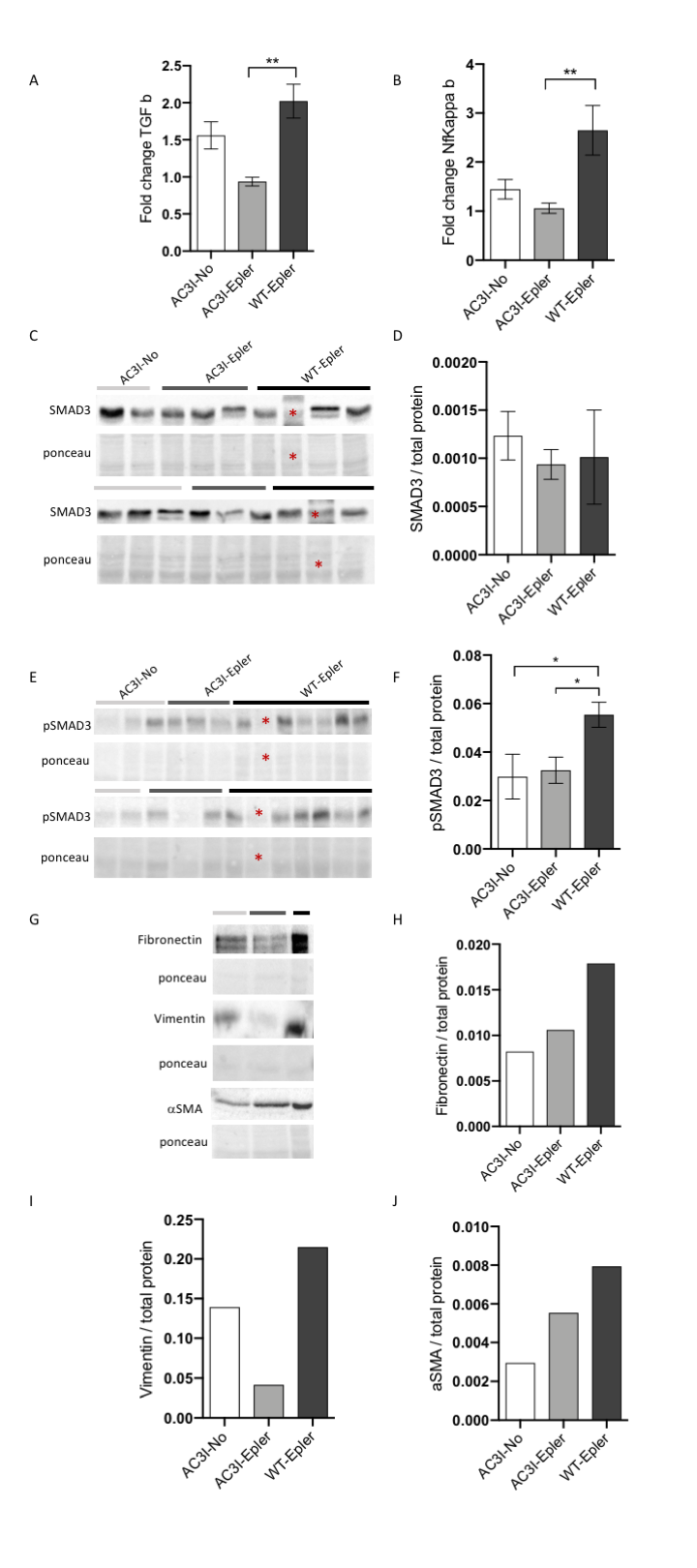


A significant increase in TGF-β (A), and Nf-κb mRNA (B) is seen in WT-Epler compared to AC3I-Epler. TGF-β: AC3I-No n=14, AC3I-Epler n=9, WT-Epler n=7. NfKb: AC3I-No n=13, AC3I-Epler n=12, WT-Epler n= 9. Western blot result (C) ,and analysis of SMAD3 (D) on mice from all three groups. AC3I-No n=5, AC3I-Epler n=6, WT-Epler n=7. Red asterisk indicates degraded samples which were left out of the analysis. Western blot results (E) and analysis of pSMAD3 (F) on mice from all three groups. AC3I-No n=5, AC3I-Epler n=6, WT-Epler n=12. Red asterisk indicates degraded samples which were left out of the analysis. Graphs (F) show a significant reduction of pSMAD3 in AC3I mice regardless of Eplerenone treatment. Western blot results (G) and quantification of pooled (n=5 per group) samples from all three groups for fibronectin, vimentin and αSMA (α smooth muscle actin). Quantification of fibronectin (H) shows the highest level in WT-Epler mice. Vimentin (I) also shows the highest level in WT-Epler mice,and the lowest in AC3I-Epler mice. αSMA (J) again shows the highest level in WT-mice. One-way ANOVA, * = p< 0.05; ** = p< 0.01

# 2 Supplemental table

Supplemental Table 1. Fibrosis in percentage at week 12

|  | **AC3I-No** | **AC3I-Epler** | **WT-Epler** |
| --- | --- | --- | --- |
| N | 15 | 13 | 13 |
| Total fibrosis | 4.26 ± 0.72 | 3.22 ± 0.23 | 4.67 ± 0.76 |
| Patchy fibrosis | 2.24 ± 0.62 | 1.25 ± 0.15 | 3.02 ± 0.69 |
| Interstitial fibrosis | 2.58 ± 0.28 | 2.27 ± 0.18 | 2.35 ± 0.29 |
|  | **GLS < -15** | **GLS > -15** |  |
| N | 11 | 28 |  |
| Total fibrosis | 2.75 ± 0.97 | 4.58 ± 2.60 |  |
| Patchy fibrosis | 0.94 ± 0.59 | 2.67 ± 2.35 * |  |
| Interstitial fibrosis | 2.00 ± 0.71 | 2.58 ± 1.00 |  |
|  | **No VT/VF** | **VT/VF** |  |
| N | 35 | 6 |  |
| Total fibrosis | 3.81 ± 0.36 | 5.45 ± 1.34 |  |
| Patchy fibrosis | 1.87 ± 0.30 | 3.89 ± 1.27 * |  |
| Interstitial fibrosis | 2.34 ± 0.15 | 2.78 ± 0.51 |  |

All values are ± SEM; Standard error of the mean. GLS; global longitudinal strain. VT ventricular tachycardia. VF; ventricular fibrillation. One-way ANOVA, * p< 0.05 compared to WT-Epler.

Students t-test * p<0.05 compared to GLS < -15, or no VT/VF
